# UP3005, a Botanical Composition Containing Two Standardized Extracts of *Uncaria gambir* and *Morus alba*, Improves Pain Sensitivity and Cartilage Degradations in Monosodium Iodoacetate-Induced Rat OA Disease Model

**DOI:** 10.1155/2015/785638

**Published:** 2015-02-23

**Authors:** Mesfin Yimam, Young-Chul Lee, Tae-Woo Kim, Breanna Moore, Ping Jiao, Mei Hong, Hyun-Jin Kim, Jeong-Bum Nam, Mi-Ran Kim, Jin-Sun Oh, Sabrina Cleveland, Eu-Jin Hyun, Min Chu, Qi Jia

**Affiliations:** ^1^Unigen, Inc., 3005 1st Avenue, Seattle, WA 98121, USA; ^2^Unigen, Inc., No. 450-86, Maebong-Ro, Dongnam-Gu, Cheonan-Si, Chungnam 330-863, Republic of Korea

## Abstract

Osteoarthritis (OA) is a multifactorial disease primarily noted by cartilage degradation in association with inflammation that causes significant morbidity, joint pain, stiffness, and limited mobility. Present-day management of OA is inadequate due to the lack of principal therapies proven to be effective in hindering disease progression where symptomatic therapy focused approach masks the actual etiology leading to irreversible damage. Here, we describe the effect of UP3005, a composition containing a proprietary blend of two standardized extracts from the leaf of *Uncaria gambir* and the root bark of *Morus alba*, in maintaining joint structural integrity and alleviating OA associated symptoms in monosodium-iodoacetate- (MIA-) induced rat OA disease model. Pain sensitivity, micro-CT, histopathology, and glycosaminoglycans (GAGs) level analysis were conducted. Diclofenac at 10 mg/kg was used as a reference compound. UP3005 resulted in almost a complete inhibition in proteoglycans degradation, reductions of 16.6% (week 4), 40.5% (week 5), and 22.0% (week 6) in pain sensitivity, statistically significant improvements in articular cartilage matrix integrity, minimal visual subchondral bone damage, and statistically significant increase in bone mineral density when compared to the vehicle control with MIA. Therefore, UP3005 could potentially be considered as an alternative therapy from natural sources for the treatment of OA and/or its associated symptoms.

## 1. Background

Osteoarthritis (OA) is a multifactorial disease that affects the entire joint structure and is characterized by cartilage destruction and loss, degeneration of soft tissues, localized bony hypertrophy including subchondral thickening and osteophyte formation, varying degrees of synovitis, and thickening of the joint capsule [1].

Over the years, progresses have been made to address the key detrimental factors driving the development and progression of OA [2]. The compilation of evidences from augmented sources suggests that osteoarthritis is no longer considered as a “wear and tear” degenerative disease anticipated to happen as a consequence of aging or no longer considered to be a “noninflammatory” form of arthritis. Acknowledging the advancement of modern imaging such as MRI, for instance, the synovial membrane inflammation, has been shown to be correlated with high prevalence to the severity and progression of OA and was believed to be the primary cause of pain [3, 4]. Similarly, an indication of its multifactorial complexity, immunological changes such as infiltration of B cells in the synovium and activation of T cells have also been documented [5, 6].

Substantial reports have shown the elusive nature to specifically single out an etiology for OA which indicates the intertwined existence of multiple factors involving mechanical and molecular events in the progression of the disease. It is cumbersome to pinpoint exactly when and where the disease originated as patients seek help at the later stage of the disease after a significant structural damage has already occurred; nevertheless, the strong correlation among synovitis, cartilage, and meniscus degradation has been described as part of a vicious circle perpetuating OA [7]. Although cartilage destruction is the main event in defining osteoarthritis, the degradation of type II collagen is the fundamental incident that determines the irreversible progression of osteoarthritis disease in association with inflammation.

Given these associations and significance, the application of a therapy with anti-inflammatory activity and conceivable cartilage degradation protection capability will certainly have a merit of use in relieving the pain and maintaining matrix integrity in these patients.

Nevertheless, present-day management of OA is inadequate due to the lack of primary therapies proven to be effective in hindering disease progression. The current approach which focuses mainly on curtailing the sensitivity of disease associated pain will only mask the actual etiology leading to irreversible damage to the joint structure. While intra-articular injection of corticosteroids, hyaluronic acid, and oral or topical nonsteroidal anti-inflammatory drugs (NSAIDs) has most frequently been used to relieve pain quicker in OA patients, glucosamine and chondroitin have also showed delayed but measurable outcome on pain and improved function in OA patients. In fact, previously, glucosamine sulfate and chondroitin sulfate were recommended by the Osteoarthritis Research Society International (OARSI) as possible structural modifying agents in hip and knee OA [8–12]. However, the recently published OARSI guidelines downgraded these agents to “uncertain” as a symptom reliever or “not appropriate” as a disease modifying agent when used for all OA patients. Similarly, oral and transdermal opioid painkillers were graded as “uncertain.” On the other hand, topical NSAIDs are recommended as appropriate for all patients with knee-only OA and were found to be safer and better tolerated compared to oral NSAIDs [13]. These periodical changes in recommendations of use by the expert panel clearly define the uncertainty of current nonpharmacological and pharmacological modalities of therapy for OA management. Intensifying the complicated situation, many distressed patients compromise their safety by inclining more towards substandard and unregulated product sources hoping to lessen the catastrophic outcome of the disease and to improve their quality of life. As a result, the need for evidence based safe and efficacious alternatives from natural sources is more urgent now than ever before.

Although the initiating cause(s) of OA is not known, elevated levels of proinflammatory cytokines such as tumor necrosis factor-alpha (TNF-*α*), interleukin (IL-1 and IL-6), cellular component of immunology, and inducible nitric oxide synthase (iNOS) and activation of nuclear factor-kappa B (NF-*κ*B) are thought to be essential for disease pathophysiology and progression [14]. The major flavan in* Uncaria gambir*, catechin, and prenylated flavonoids and stilbenoids, from the root bark of* Morus alba *L., possess activities suggestive of benefits in OA including (i) inhibition of the activity of cyclooxygenase-2 (COX-2), lipoxygenase (5-LOX), platelets phospholipase A2 and proinflammatory cytokines tumor necrosis factor-alpha (TNF-*α*), interleukins (ILs) 1, 2, 6, 8, and 12 [15, 16] as a result of catechin; (ii) inhibition of inflammation activities [17]; (iii) suppression effect of T-cell migration; (iv) inhibition of CXCR-4-mediated chemotaxis and MEK/ERK pathway in T-cell and other immune cells [18]; (v) inhibition of nitrogen oxide (NO) production, inducible NO synthase expression, prostaglandin E2 production, and activation of NF*κ*b in macrophages [19]; and (vi) inhibition of proinflammatory mediators such as COX-2, IL-1*β*, and IL-6 and enhancing total antioxidant ability [20, 21] as a result of prenylated flavonoids and stilbenoids from* M. alba* root bark extract have been reported.

*Morus alba* L. (Moraceae), the mulberry or white berry plant, is native to northern China and has been cultivated and naturalized elsewhere, from India to the Middle East to Southern Europe and recently to the North American region. According to Pharmacopoeia of China, the root bark of* Morus* is used in traditional medicine known as Sang bai pi to drain heat from the lung reducing coughing and wheezing. This herb is also known as Pong-na-moo for cough and hypertension in Korean and Sohakuhi for skin care in Japan. In contemporary pharmacological research,* Morus alba* root bark has been reported to have antibacterial, antiviral, antioxidant, hypoglycemic, hypolipidemic, neuroprotective, antiulcer, analgesic, and anti-inflammatory activities. A variety of bioactive compounds from* Morus alba* root bark have* in vivo* and* in vitro* anti-inflammatory activity [17–21].

*Uncaria gambir* (Rubiaceae) is a climbing shrub with round branches, which traditionally is believed to strengthen teeth when chewed with piper betle leaves in Asia. Leaves of the* U. gambir* plant contain free catechins as well as polymerized catechins, tannins, which are more abundant in younger leaves as compared to older leaves. In the Republic of Korea, there are many over-the-counter (OTC) drugs that contain* U. gambir* extract, especially for dyspepsia, halitosis, vomiting, and anorexia. While* U. gambir* is used for diarrhea, vomiting, and gastritis in Japan, it is also used as a dietary supplement to support liver function and fat metabolism in the United States of America [15, 16].

Glycosaminoglycan is a major component of joint cartilage, joint fluid, and other soft connective tissues. The glycosaminoglycans (GAGs) of articular cartilage have been identified as chondroitin 6-sulphate, chondroitin 4 sulphate, dermatan sulfate, heparin, heparin sulfate, and keratin sulfate. GAGs are released from the degrading cartilage matrix in large amounts during inflammation of the joints. Changes in the levels or molecular nature of GAGs have been associated with some connective tissue diseases. For example, patients with arthritis and scleroderma have elevated concentrations of GAGs in blood and synovial fluid, and destruction of involved joints in arthritis patients correlates positively with high GAG levels in synovial fluid [22]. Histochemical and biochemical studies of cartilage from arthritic joints have shown a significant decrease in the GAG content and that the decrease is approximately proportional to the severity of the disease [23].

Multiple animal models have been developed and utilized to study the pathogenesis of OA and to evaluate the effectiveness of novel therapeutic agents with limited success. It is imperative to select an animal model that consistently reproduces the joint pathology and pain associated with the disease. In this regard, we employed a minimally invasive monosodium-iodoacetate- (MIA-) induced OA model due to its robust induction and reproducibility. Monosodium-iodoacetate (MIA) is an inhibitor of glyceraldehyde-3-phosphate dehydrogenase activity shown to induce chondrocyte death and hence reproduces cartilage lesions with loss of proteoglycan matrix and functional joint impairment similar to human OA [24].

Here, we describe the elucidation of use of UP3005, a composition containing a proprietary blend of two standardized extracts from leaf of* Uncaria gambir* and root bark of* Morus alba*, in maintaining joint structural integrity and alleviating OA associated symptoms in MIA-induced rat OA disease model.

## 2. Materials and Methods

### 2.1. Individual Materials

*Uncaria gambir* leaves were collected in Gunung Malintang area of Sumatra, Indonesia. The dried leaves of* U. gambir* (2 kg) were extracted two times with 15-fold volume of water at 80°C for 7 hrs. The combined extraction solution was filtrated and dried under vacuum to afford 120 g of* U. gambir* extract.* Morus alba* root barks were collected in Sichuan province of China. The dried root barks of* Morus alba* (2 kg) were extracted two times with 7-fold volume of 70% aqueous ethanol at 80°C for 5 hrs. The combined extraction solution was filtrated and dried under vacuum to afford 294 g of ethanol extract powder.

### 2.2. The Composition

The composition material of UP3005 was prepared by blending two standardized extracts including* Uncaria gambir* leaf and* Morus alba* root bark, respectively, with 1 : 1 ratio. The* Uncaria gambir* leaf extract contains (+)-catechin as the major component with a content of not less than 16%. The* Morus alba* root bark extract contains stilbenoid mulberroside A, not less than 4%. The content for each individual marker compound in UP3005 was determined and quantified by HPLC method using an Agilent HPLC/PDA system with a Zorbax Eclipse XDB C18 reversed-phase column. For quantification of active marker, catechin standard was purchased from Sigma (Catalog number: 21510-4) and mulberroside A was purchased from Chengdu Biopurify Phytochemicals (Catalog number: BP0964).

### 2.3. HPLC Analysis Conditions

The following analytical method was used to determine the amount of catechin in the* Uncaria gambir* leaf extracts and mulberroside A in the* Morus alba* root extracts. An Agilent HPLC/PDA system with a C18 reversed-phase column (Zorbax Eclipse XDB, 3.5 m, 4.6 mm × 150 mm, Agilent) using a guard column of C18 cartridge (4.0 mm × 3 mm, Phenomenex) was used for the detection and quantitation of catechin and mulberroside A. Mobile phases consisted of 0.1% phosphoric acid in purified water (A) and methyl alcohol (B). The elution conditions are described as follows: elution time, 0~15 min, 12% → 18% B (v/v); 15 min~25 min, 18% B → 100% B (v/v) for cleaning with the injection volume of 10 L. The flow rate was set to 1.0 mL/min passing through the Zorbax C18 column with a column temperature of 35°C with the UV detection absorbance at 285 nm.

### 2.4. *Ex Vivo*

Articular cartilage from hock joints of rabbits (2.5 kg body weight) was removed immediately after each animal was sacrificed. The articular cartilage explants were obtained by following the method described by Sandy et al. [25]. Briefly, after the articular surfaces were exposed surgically under sterile conditions, approximately 200–220 mg articular surfaces per joint were dissected and submerged into complete medium (Dulbecco's Modified Eagle's medium (DMEM), supplemented with heat-inactivated 5% Fetal Bovine Serum (FBS); penicillin 100 U/mL; streptomycin 100 *μ*g/mL). They are then rinsed several times with the complete medium and incubated for 1 to 2 days at 37°C in a humidified 5% CO_2_/95% air incubator for stabilization. The complete medium was replaced with a basal medium (DMEM, supplemented with heat-inactivated 1% FBS, 10 mM hydroxyethyl piperazineethanesulfonic (HEPES), and penicillin 100 U/mL, streptomycin 100 *μ*g/mL). Approximately 30 mg cartilage pieces (2 × 3 × 0.35 mm/piece) were placed in 24-well plates and treated with given concentrations of test agents. After pretreatment for 1 h, 5 ng/mL of recombinant human interleukin-1 beta (rhIL-1) was added to the culture medium and further incubated at 37°C in a humidified 5% CO_2_/95% air incubator. The culture medium was collected 24 h later and stored at −20°C until assay. The amount of sulphated GAGs in the medium at the end of reaction reflecting the amount of articular cartilage degradation was determined by 1,9-dimethyl-methylene blue method using commercially available kit (the Blyscan proteoglycan and glycosaminoglycan assay) according to the instructions of the manufacturer. Additional assay was also performed at a 1 : 1 ratio of* U. gambir* and* M. alba* at 100 *μ*g/mL to compare with 200 *μ*g/mL of UP3005 for synergy, if any.

### 2.5. *In Vivo*

Male Sprague-Dawley (SD) rats weighing 160~180 g (6 weeks of age) were purchased from Orient Bio Co., Ltd. (Korea) and acclimated for one week. Rats (2/cage) were housed in a polycarbonate cage and individually identified by numbers on their tail. Individual cage was identified with a cage card indicating project number, test article, dose level, group, and an animal number. Animals were provided with fresh water and rodent chow diet # 2018s (Harlan Teklad, USA; supplied by Nara biotech, Korea)* ad libitum* and were kept in a temperature controlled conventional animal room (23.1°C) on a 12-hour light-dark cycle. One day before disease induction, animals (body weight 237~267 g) were randomized into four groups of 10 rats per group as G1 = normal, G2 = vehicle (0.5% CMC-Na solution), G3 = Diclofenac (10 mg/kg), and G4 = UP3005 (500 mg/kg) (Lot #051M0191V) and orally gavaged with respective treatment. Treatment started a day before MIA injection. Anesthetized rats were injected with 0.8 mg of MIA in 50 *μ*L saline solution into the intra-articular pocket of left knee joint using 26 G needle an hour after the second dose of treatment. Normal control rats were injected with an equal volume of saline. MIA powder was stored in a desiccator and solutions were prepared fresh at time of injection. Paw withdrawal thresholds as a result of constant pressure applied perpendicular to the hind paw as a measure of pain sensitivity were taken once a week using Randall-Salitto Anesthesiometer (IITC, USA) and treatment lasted for 6 weeks. Body weights were measured once a week to calculate the respective weekly dosage of each group.

Once the in-life study was concluded, animals were asphyxiated with CO_2_ and the femorotibial joint was dissected out for further Micro-CT and histopathology analysis. Six prototype animals in each group were subjected to histomorphometry for evaluation of structural alterations of their articular cartilage surface and subchondral bone architecture of both femur and tibia of the knee joint using Micro-CT scan (Inveon, Siemens Medical Solutions USA, Inc.) at KBSI (Korea Basic Science Institute).

For histopathological examination, the knee joints, including the patella and joint capsule, were kept in the 10% formalin for 72 hrs. The fixed specimens were then decalcified with Calci-Clear Rapid for 1 and a half days and embedded in paraffin. Standardized 5 *μ*m serial sections were obtained at the medial and lateral midcondylar level in the sagittal plane and were stained with hematoxylin and eosin (HE) and Safranin O-fast green to enable evaluation of proteoglycan content. A modified Mankin system [26] was used to score structural and cellular alterations of joint tissues as the results of disease progression and/or treatment efficacy. The histological analysis was conducted at the College of Veterinary Medicine, Kyungpook National University, and slides were examined by Professor Park, DVM, Ph.D.

All animal experiments were conducted according to institutional guidelines congruent with the guide for the care and use of laboratory animals, which were reviewed and approved by the Institutional Animal Care and Use Committees (IACUC) (Approval number UIK21304).

## 3. Statistical Analysis

Data were analyzed using Sigmaplot (Version 11.0). The results are represented as mean ± one SD. Statistical significance between groups was calculated by means of single factor analysis of variance followed by a paired *t*-test. *P* values less than or equal to 0.05 (*P* ≤ 0.05) were considered as significant. When normality test failed, for nonparametric analysis, data were subjected to Mann-Whitney sum ranks for *t*-test and Kruskal-Wallis one-way analysis of variance on ranks for ANOVA.

## 4. Results

**Figure 1:**
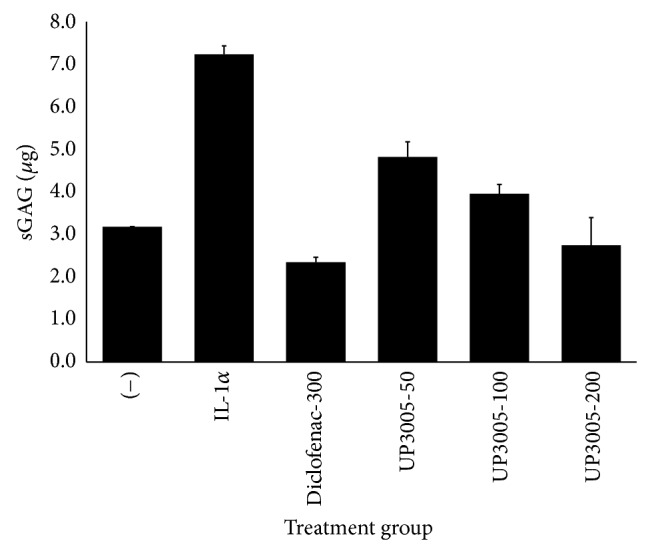
Effect of UP3005 composition on GAG releasing. Cartilage explants were incubated for 24 h in DMEM with 1% heat-inactivated FBS. Each set of data represents the means ± SD (−): control cultured medium without IL-1*α*; IL-1*α*: cultured medium treated with IL-1*α* (5 ng/mL); Diclofenac-300: cultured medium treated with IL-1*α* plus 300 *μ*g/mL of Diclofenac; UP3005-50/100/200: cultured medium treated with IL-1*α* plus UP3005 at doses of 50/100 or 200 *μ*g/mL.

*Ex vivo*, rabbit cartilage explants were cultured for 24 hrs with IL-1*α* (5 ng/mL) in the absence or presence of test agents to examine the protective effects on proteoglycans (PG) degradation. Results were expressed as *μ*g GAG released into the medium. When the rabbit cartilage explants were treated with IL-1*α*, the amount of released GAG into the culture medium increased significantly when compared to the vehicle group. As shown in Figure 1, UP3005 composition reduced IL-1*α* mediated degradation of PG in a concentration dependent manner (*P* < 0.05). UP3005 at 200 *μ*g/mL almost totally inhibited the PG degradation. The level of proteoglycans in culture medium from UP3005 at 200 *μ*g/mL is similar to that of negative control group without addition of IL-1*α*. As determined by Colby's equation, greater inhibition was also observed from the current assay when the culture medium was treated by the composition UP3005 than* U. gambir* or* M. alba* alone (Table 1).

One of the main cardinal symptoms of OA (i.e., pain) was evidenced a week following model induction. As depicted in Table 2, rats with an intra-articular injection of MIA without treatment showed progressive increase in pain sensitivity as exhibited by the mean pain sensitivity values. In contrast, rats treated orally with a daily dose of 500 mg/kg of UP3005 showed statistically significant reductions in pain sensitivity after 4 weeks of oral treatment. Reductions of 16.6%, 40.5%, and 22.0% in pain sensitivity were observed for rats treated with UP3005 (500 mg/kg) at week 4, week 5, and week 6, respectively. Diclofenac showed significant 20.8% reduction of pain sensitivity as of week 5 and 18.2% was recorded for week 6.

**Figure 2:**
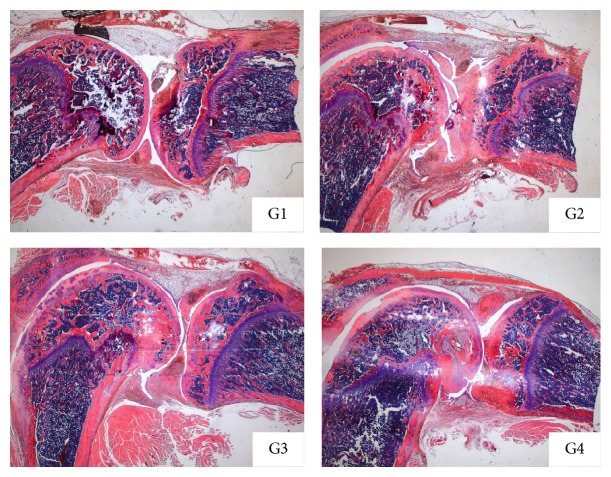
Histopathological findings. G1: normal, G2: vehicle G3: 10 mg/kg of Diclofenac, and G4: 500 mg/kg of UP3005.

In agreement with the pain sensitivity reduction data, statistically significant improvements in articular cartilage matrix integrity were shown as reflected by the total Mankin score for animals treated with UP3005 at a dose of 500 mg/kg (Table 3 and Figure 2). However, a positive trend without statistical significance in changes of structure, cellular abnormality, and matrix integrity was also observed from the positive control, Diclofenac, when compared to vehicle control (Table 3 and Figure 2). All the sections obtained from the vehicle treated group with MIA injection showed irregular surfaces with focally extensive area of cartilage loss and degeneration with ulceratic necrosis on the femoral and tibial sides together with focal collapse, infiltrated inflammatory cells, and extensive areas of fibrosis that replaced adjacent bone trabeculae. In Safranin O staining, articular cartilage of UP3005 revealed the reduction or loss of staining intensity in the superficial and middle zones with necrotic degeneration. In contrast, none of the sections obtained from control group showed such changes.

**Figure 3:**
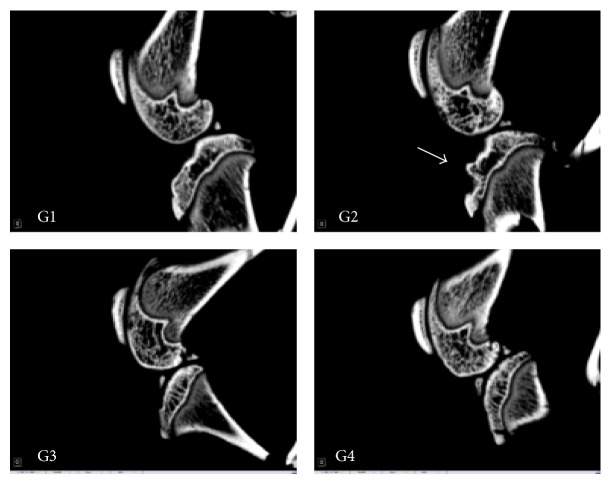
Micro-CT (sagittal plane). G1: normal, G2: vehicle, G3: Diclofenac 10 mg/kg, and G4: UP3005 500 mg/kg.

**Figure 4:**
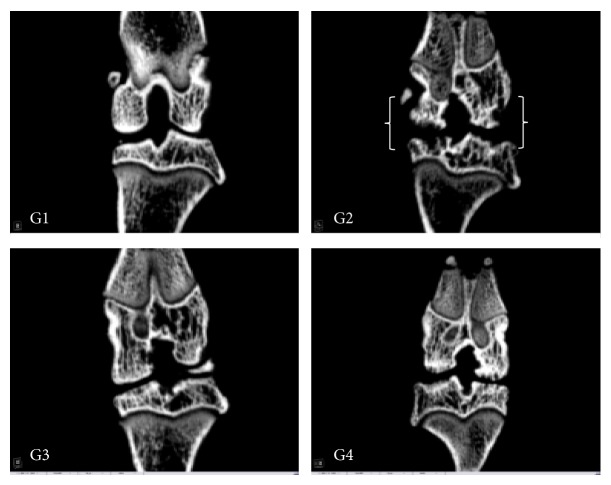
Micro-CT (coronal plane). G1: normal, G2: vehicle, G3; Diclofenac 10 mg/kg, and G4: UP3005 500 mg/kg.

Substantiating the above findings, it was noted that rats treated with Diclofenac and UP3005 showed minimal visual subchondral bone damage compared to the vehicle control with MIA, which experienced extensive regional damage as seen on the sagittal and coronal femorotibial joint *μ*-CT imaging (Figures 3 and 4). In fact, rats treated with 500 mg/kg of UP3005 showed statistically significant increase in bone mineral density and a strong tendency of improved trabecular bone volume, turnover, and thickness when compared to the vehicle control treated group (Table 4).

## 5. Discussion

Previously, significant reports have demonstrated the possible beneficial use of* U. gambir* and* M. alba* in OA. For instance, the major flavan in* gambir*, catechin, has been shown to inhibit the activity of cyclooxygenase-2, lipoxygenase, platelet phospholipase A2, proinflammatory cytokines such as tumor necrosis factor-alpha (TNF-*α*), and multiple interleukins (ILs), that is, interleukins 1, 2, 6, 8, and 12 [15]. Similarly, a variety of bioactive compounds from* Morus alba* root bark have shown* in vivo* or* in vitro* anti-inflammatory activity. For example, suppression of T-cell migration by crude extract and ethyl acetate fractions of root bark, inhibition of chemokine receptor type 4- (CXCR-4-) mediated chemotaxis and mitogen-activated protein kinases/extracellular signal-regulated kinases (MEK/ERK) pathway in T-cell and other immune cells [18], inhibition of nitrogen oxide (NO) production, reduction of inducible NO synthase expression, inhibition of prostaglandin E2 production and activation of NF*κ*b in macrophages by oxyresveratrol [19], and inhibition of both NO production and iNOS as well as reduction of proinflammatory mediators such as COX-2, IL-1*β*, and IL-6 by total flavonoids from the root bark [20] and by prenylated flavonoids including morusin [21] from* M. alba* were reported.* In vivo*, oxyresveratrol and mulberroside A have been reported with anti-inflammatory effect on carrageenan-induced paw edema model in rats at dosages of 7.5 mg/kg and 50 mg/kg, respectively [19]. Hence, the hypothesis of formulating these historically well-known plant materials into a well-defined specific composition for indication of OA associated symptoms and/or disease modification was described in this report.

Historically, histopathological analysis of the articular cartilage, synovial membrane, and joint capsule has been the most frequently used tool to assess the degree of severity of OA or to evaluate changes as a result of therapeutic interventions [27]. In this regard, we developed the MIA-induced OA disease model in rats and employed to evaluate the potential anti-inflammatory and/or cartilage degradation protection activity of UP3005, a composition containing a proprietary blend of two standardized extracts from leaf of* Uncaria gambir* and root bark of* Morus alba*. An intra-articular injection of MIA inhibits glyceraldehyde-3-phosphate activity in chondrocytes which results in a rapid onset of model development by disruption of glycolysis culminating with cell death [28]. As the disease progresses, finding corresponding histopathological and morphological changes of the articular cartilage and surrounding structure of the joint closely resembling human OA is a common phenomenon.

A significant progressive suppression in pain sensitivity in diseased rats treated with an oral dose of UP3005 for 6 weeks was observed; this finding was not a surprise knowing the fact that UP3005 possesses those qualities and contributed by active flavonoids extracted from both* gambir* and* Morus* plants which have a strong anti-inflammatory activity as described above.

These alleviations of disease associated pain symptoms were also shared by the common NSAID, Diclofenac, as expected.

Similarly, we have observed limited loss, degeneration, or necrosis of chondrocytes, smoother articular cartilage surface, deeper and uniform stain of intracellular matrix, and close to normal contour of the subchondral bone which suggest the significant improvement in maintaining the structural integrity of the joint for the rats treated with the composition UP3005. It has been reported that normal chondrocytes do not divide and regulate the balance between catabolism and anabolism in cartilage [29]; however, when their surface receptors stimulated by mechanical stress, aging factors, and proinflammatory mediators, such as TNF-alpha (TNF-*α*) or interleukin (IL-1*β*), this will lead to induction of the NF-*κ*B signaling pathway which triggers the secretion of several matrix-degrading proteinases [30]. Moreover, the NF-*κ*B pathway could also be activated by synoviocytes upon stimulation by similar factors such as mechanical stress or cytokines (TNF-*α* and IL-1*β*) which subsequently produce additional several matrix metalloproteinases (MMPs), a disintegrin and metalloproteinase with thrombospondin motifs (ADAMPTS), apoptotic molecules (COX2, NO, and prostaglandin E2), chemokines (IL-8), and cytokines (TNF-*α*, IL-1*β*, and IL-6) ensuing cartilage destruction and synovium membrane inflammation. Therefore, these particular beneficial activities of UP3005 in OA could be in part (1) by protection of cartilage degradation as shown by its greater inhibition of sGAG release possibly by inhibiting multiple matrix-degrading proteinases and/or (2) through interventions at the inflammatory process by disrupting the vicious cycle that links cartilage degradation to the synovitis possibly by inhibiting primary proinflammatory mediators. These mediations may potentially hinder the recurrent attack of the joint by either the degradants from the cartilage or proinflammatory cytokines from the synovial membrane which ultimately provides pain relief and improved joint mobility and stability. Moreover, it can also be inferred from the expected and experimental inhibition values that combining the two extracts (gambir and morus) could provide a greater protection to proteoglycan degradation than either of the components given alone which further strengthen the significance of the composition.

As the Mankin score attests, a positive trend without statistical significance in changes of structure, cellular abnormality, and matrix integrity was observed for the positive control, Diclofenac, when compared to vehicle. This lack of significant cartilage protection observed by Diclofenac further signifies the limitation of NSAIDs for prolonged usage in OA patients besides symptomatic pain relief. Paralleling our findings, significant analgesic activity [31, 32] but limited protection of cartilage injury and proteoglycan degradation as a result of orally administered Diclofenac were reported [33]. Normal structure of the articular cartilage, subchondral bone of both tibia plateaus and femoral bone, and the surrounding joint structure appeared intact in the control rats. In contrast, various degrees of histopathological changes including cellular degeneration and disorganization of the articular cartilage chondrocytes, depletion and collapse of the intracellular matrix, articular surface irregularities, osteophyte remodeling, and fibrillation of the subchondral bone were observed for MIA-injected rats treated with vehicle. These changes were the most common findings in human OA biology [1]. A number of currently used OA supplements or drugs under development have used the MIA model to evaluate the efficacy of compound in query; in fact, substantiating our findings, recently Lee et al. [34] have shown suppression of articular cartilage damage induced by MIA after 50 days of daily oral treatment of frequently used OA supplement and glucosamine/chondroitin sulfate, at a dose of 250 mg/kg.

Additionally, as seen in the sagittal and coronal microfocal computed tomography images, in rats treated with a daily dose 500 mg/kg of UP3005 for 6 weeks, visually, the cross-talk between articular cartilage and osteoclast activity directly underneath the subchondral trabecular bone has seemed mitigated. This mitigation in turn could have reduced the dynamic changes in the subchondral bone plate which would have caused increased porosity, thinning, and erosion of the bone otherwise. Regardless of the initial direction where OA impacts the surrounding articular structure, changes in the overlying articular cartilage [35, 36] and increase in subchondral bone turnover [37, 38] are primary causes for extensive interactions between cartilage and bone during disease progression to trigger one another. Hence, the minimal damage observed in the current Micro-CT imaging could be the result of beneficial interception of UP3005 at either the cartilage or the subchondral initiation stage or both leading to reduction of severe bone remodeling or erosion. In supplement, the significant increase in bone density volume observed in UP3005 treated rats could signal additional benefit of the composition in minimizing the risk of bone fracture and/or osteoporosis.

It is a fundamental necessity for an anti-OA therapeutic intervention to achieve its maximum potential in modifying the progression of a disease and/or alleviating associated symptoms. To attain these central objectives, it is desirable for a compound to impact all articular components and curve the dynamics of cartilage degradation, subchondral bone sclerosis, and synovial inflammation. The data depicted here clearly favors the benefit of using UP3005 to prevent cartilage degradation and to mitigate an inflammation process at the apex of the cascade or by intervening at some point in the metabolic and structural modification linkage to hamper progression of the disease. However, safety and the direct effect of the composition on the integral part of the disease such as cartilage, bone, and synovial membrane with associated biomarkers need further investigation.

## 6. Conclusions

In summary, we evaluated the efficacy of orally administered UP3005, a composition containing a proprietary blend of two standardized extracts from the leaf of* Uncaria gambir* and the root bark of* Morus alba*, in monosodium-iodoacetate- (MIA-) induced osteoarthritis disease model in rats. Documenting the pain sensitivity, Micro-CT, and histopathology data from this study, UP3005 could potentially be considered as an alternative therapy from natural sources for the treatment of OA and/or its associated symptoms. However, further preclinical and human clinical studies are necessary to address the association of these findings with specific OA related diagnostic biomarkers as indication of disease progression or repression influenced by UP3005.

## Figures and Tables

**Table 1:** Possible synergistic activity of *U*. *gambir *and *M*. *alba *in protection in composition UP3005.

Sample	Dose (*μ*g/mL)	% inhibition	Remark
UP3005	200	71.8	Expected value
		88.5	Experimental value
*U*. *gambir *	100	32.6	—
*M*. *alba *	100	58.2	—

Rabbit cartilage explants were cultured for 24 hours with rhIL-1*α* (5 ng/mL) in the absence or presence of a *gambir* extract, *Morus* extract, or UP3005 at the disclosed doses to examine the potential protective effect on proteoglycan degradation. Whether a synergistic effect was present was calculated by using Colby's formula [39]. Expected value: theoretically calculated inhibition value based on Colby's formula for synergy; experimental value: inhibition observed at 200 *μ*g/mL of UP3005; synergy = experimental value ≥ expected value.

**Table 2:** Percent changes of pain sensitivity by composition UP3005 in MIA-induced OA rat model.

Group/dose	Week						
	0	1	2	3	4	5	6
G1 = normal							
Mean	174.53	172.53	174.67	175.40	171.40	166.07	163.60
SD	29.51	6.53	13.90	23.95	39.09	19.86	9.32
%	100.00	98.85	100.08	100.50	98.20	95.15	93.74
G2 = vehicle							
Mean	173.23	158.07	151.90	160.07	143.10	121.03	122.07
SD	18.04	15.08	42.97	16.12	23.00	12.77	15.17
%	100.00	91.24	87.69	92.40	82.61	69.87	70.46
G3 = Diclofenac (10 mg/kg)							
Mean	173.20	146.37	155.67	162.90	150.90	146.23	144.23
SD	15.61	27.86	23.85	28.90	19.62	22.73	27.57
%	100.00	84.51	89.88	94.05	87.12	84.43	83.28
G4 = UP3005 (500 mg/kg)							
Mean	173.33	168.13	178.87	174.37	166.90	170.00	148.93
SD	17.44	22.68	57.83	26.48	27.17	21.13	26.12
%	100.00	97.00	103.19	100.60	96.29	98.08	85.92
*P* values versus vehicle G2							
G1	0.9311	0.0226	0.1535	0.2446	0.1907	0.0041	0.0000
G3	0.9965	0.2626	0.8120	0.7905	0.4255	0.0085	0.0428
G4	0.9901	0.2600	0.2532	0.1655	0.0491	0.0000	0.0135

Paw withdrawal thresholds as a result of constant pressure applied perpendicular to the hind paw as a measure of pain sensitivity were taken once a week using Randall-Salitto Anesthesiometer. Applied pressures were translated into grams by the Anesthesiometer. *N* = 10 rats/group.

**Table 3:** Modified Mankin scoring system for histopathological findings for MIA-induced rats treated with UP3005 orally.

Group/dose	Structure (0~6)	Cellular abnormality (0~3)	Safranin O staining (0~4)	Total Mankin score
Normal	0	0	0	0
Vehicle	2.78 ± 1.79	1.78 ± 0.44	2.67 ± 1.32	7.22 ± 3.19
Diclofenac (10 mg/kg)	1.9 ± 1.45	1.3 ± 0.67	1.7 ± 0.82	4.9 ± 2.69
UP3005 (500 mg/kg)	1.0 ± 0^*^	1.4 ± 0.52	1.3 ± 0.95^*^	3.7 ± 1.33^*^

Structure (0~6)—0 = normal, 1 = irregular surface, including fissures into the radial layer, 2 = pannus, 3 = absence of superficial cartilage layers, 4 = slight disorganization (an absent cellular row and some small superficial clusters), 5 = fissures into the calcified cartilage layer, and 6 = disorganization (chaotic structure, clusters, and osteoclastic activity); cellular abnormalities (0~3)—0 = normal, 1 = hypercellularity, including small superficial clusters, 2 = clusters, and 3 = hypocellularity; matrix staining (0~4)—0 = normal/slight reduction of staining, 1 = staining reduced in the radial layer, 2 = staining reduced in the interterritorial matrix, 3 = staining reduced in pericellular matrix, and 4 = staining absent. *N* = 10 rats/group, ^*^ *P* ≤ 0.05 versus vehicle.

**Table 4:** Change of BMD and architecture in MIA-induced rats treated with UP3005 orally.

	Normal	Vehicle	Diclofenac (10 mg/kg)	UP3005 (500 mg/kg)
BMD (mg/cm^3^)	1023.5 ± 8.3	881.9 ± 64.0	982.3 ± 19.4^*^	996.1 ± 11.6^*^
BV/TV (%)	74.76 ± 2.60	68.24 ± 5.03	71.89 ± 5.43	70.84 ± 5.92
BS/BV (1/mm)	7.22 ± 0.65	8.76 ± 1.13	7.82 ± 0.98	8.03 ± 0.74
Tb. Th (mm)	0.28 ± 0.02	0.23 ± 0.03	0.26 ± 0.03	0.25 ± 0.02
Tb. Sp (mm)	0.094 ± 0.006	0.107 ± 0.012	0.100 ± 0.015	0.103 ± 0.02

Data represented as mean ± SD. ^*^ *P* < 0.05 (versus vehicle). BV/TV: total trabecular bone volume/total tissue (bone + marrow) volume; BS/BV: trabecular bone surface/trabecular bone volume; Tb. Th: trabecular thickness; Tb. Sp: trabecular spacing. *N* = 10 rats/group.
